# Autophagy is essential for anti-*Wolbachia* drug efficacy in *Brugia malayi* and insect cells

**DOI:** 10.3389/fmicb.2026.1771755

**Published:** 2026-03-16

**Authors:** Anfal Yousef, Ellen K. G. Masters, Yang Wu, W. David Hong, Paul M. O’Neill, Joseph D. Turner, Steve A. Ward, Mark J. Taylor

**Affiliations:** 1 Centres for Drug and Diagnostics and Neglected Tropical Diseases, Department of Tropical Disease Biology, Liverpool School of Tropical Medicine, Liverpool, United Kingdom; 2 The Robert Robinson Laboratories, Department of Chemistry, The University of Liverpool, Liverpool, United Kingdom

**Keywords:** autophagy, chemotherapy, lymphatic filariasis, onchocerciasis, *Wolbachia*

## Abstract

**Introduction:**

Onchocerciasis and lymphatic filariasis (LF) cause a significant global public health burden with more than 900 million individuals at risk and over 60 million people living with symptomatic manifestations caused by filarial diseases. Due to the importance of *Wolbachia* for the survival of adult filarial nematodes, anti-*Wolbachia* therapy has been validated as a safe macrofilaricidal treatment for LF and onchocerciasis. The A-WOL consortium was established with the goals of defining registered anti-*Wolbachia* antibiotics, as well as developing new drugs effective in a regimen of 7 days or less. We have previously shown autophagy has a core role in the regulation of *Wolbachia* populations across a diverse range of associations. In this study, we investigated the role of autophagy in the efficacy of anti-*Wolbachia* drugs.

**Methods:**

Autophagic flux was assessed in response to broad-spectrum anti-*Wolbachia* antibiotics (doxycycline, rifampicin, moxifloxacin, and sparfloxacin) and A-WOL candidate compounds (flubentylosin [TylAMac], AWZ1066S, and fusidic acid), compared with antibiotics with no activity against *Wolbachia* (levofloxacin, ciprofloxacin, amoxicillin, and streptomycin). Autophagy was quantified through LC3B-I to LC3B-II conversion and p62 degradation in insect cell lines (C6/36 and Sf9) and in *Brugia malayi*. The role of autophagy was evaluated using early- and late-stage inhibitors. The generation of ROS was measured to assess its contribution to autophagy activation. Finally, the viability of purified extracellular *Wolbachia* following drug exposure was determined using live/dead staining and reinfection assays.

**Results:**

All effective anti-*Wolbachia* compounds induced autophagic flux in insect cells and *B. malayi*, whereas ineffective antibiotics did not. Autophagy activation occurred in the absence of *Wolbachia*, was restricted to insect and nematode systems, and was not preceded by the generation of ROS. Only concentrations that induced autophagy resulted in effective *Wolbachia* depletion (of >90%), the empirical threshold of delivering the desired macrofilaricidal activity, and autophagy inhibition reduced the efficacy of the drugs. Exposure of purified extracellular *Wolbachia* to anti-*Wolbachia* drugs showed no impact on their viability, indicating that host processes are required for depletion.

**Discussion:**

These results demonstrate the critical role of host autophagy in anti-wolbachia drug activity and a previously unrecognised host-directed mechanism in insect cells and *B. malayi*.

## Introduction

Onchocerciasis and lymphatic filariasis (LF) are neglected tropical diseases caused by a family of filarial nematodes. Severe clinical manifestations of lymphatic filariasis include lymphoedema of the limbs and swelling of the scrotal sac, both of which can be exacerbated by secondary bacterial infections leading to acute dermatolymphangioadenitis. Onchocerciasis can cause skin disease associated with severe itching and depigmentation and ocular lesions that can lead to visual impairment and blindness (Taylor et al., 2010). Three anthelminthic drugs are currently available to target lymphatic filariasis: albendazole, diethylcarbamazine citrate (DEC) and ivermectin, with only ivermectin used against onchocerciasis. Since these drugs are microfilaricidal only, both diseases rely on annual mass drug administration programs lasting the length of the reproductive lifespan of the female worms (World Health Organization, 2025). This, alongside contraindications and reports of resistance to ivermectin, led to the WHO highlighting the importance of alternative drug regiments (World Health Organization, 2017). One validated approach for delivering macrofilaricidal therapy (adult parasite death) has come from targeting *Wolbachia*, an essential intracellular bacterium with a mutualistic association with most medically important filarial nematodes.

*Wolbachia* are found in the lateral chord of both male and female worms, as well as the reproductive system and embryonic stages in the uterus of females (Slatko et al., 2010). These bacteria are necessary for normal worm development, growth, and embryogenesis and their depletion with doxycycline leads to the permanent sterility of adult worms prior to their death (Hoerauf et al., 2008; Taylor et al., 2005a). Both 4- and 6-week courses of doxycycline (200 mg/day) were found to have significant macrofilaricidal activity 24 months after treatment, and an 8-week course after 14 months (Taylor et al., 2005b; Debrah et al., 2006, 2007). Despite this significant effect, doxycycline is unsuitable for implementation in mass drug administration (MDA) programs due to the logistics of delivering a relatively long course of treatment and its contraindication in pregnancy, during breastfeeding and in children below the age of 8 years old (Taylor et al., 2013a).

Other antibiotics have also shown to be active against *Wolbachia*, though there is variation in their efficacies. Rifampicin, of the rifamycin class, has been shown as the most effective registered antibiotic against *Wolbachia* (Fenollar et al., 2003; Townson et al., 2006; Aljayyoussi et al., 2017). Of the tetracyclines, doxycycline, tetracycline and minocycline all show good efficacy against *Wolbachia*, with minocycline showing the greatest potency (Specht et al., 2008; Johnston et al., 2014; Sharma et al., 2016). There is more variability within the fluoroquinolone class. Sparfloxacin and moxifloxacin are effective at reducing *Wolbachia* loads whereas levofloxacin is not (Fenollar et al., 2003; Johnston et al., 2017). Another fluoroquinolone, ciprofloxacin, has repeatedly been reported to have little or no effect on *Wolbachia* load (Hoerauf et al., 2000; Hermans et al., 2001; Fenollar et al., 2003). Several candidate compounds with anti-*Wolbachia* activity have also been identified through high-throughput screening by the A·WOL consortium, including AWZ1066S which achieved its anti-*Wolbachia* properties after just 1 day drug exposure (Johnston et al., 2017; Clare et al., 2019; Hong et al., 2019).

There is evidence that autophagy is involved in the regulation of *Wolbachia* populations in filarial nematodes and insect hosts (Taylor et al., 2013b; Voronin et al., 2012). While *Wolbachia* and the filarial nematode host share a mutual symbiotic relationship, *Wolbachia* may still be recognized as a foreign “pathogen” by the host immune system (Taylor et al., 2013b). Voronin et al. (2012) have examined this relationship by chemically and genetically inducing and suppressing autophagy, which resulted in a decrease and increase of *Wolbachia* load, respectively. Furthermore, the researchers described the observed bacterial elimination of *Wolbachia* following nematode autophagic activation to have future potential in antibiotic treatment against filarial diseases (Taylor et al., 2013b; Voronin et al., 2012).

Autophagy is a conserved eukaryotic degradation pathway within cells and has multiple important functions such as quality control of proteins, homeostasis and regulating the removal of damaged proteins and organelles (Levine and Kroemer, 2019). Macroautophagy, herein referred to as autophagy, is one of three types of autophagy and is mediated by autophagosomes (Mizushima, 2007). The process begins with a double membrane, called a phagophore, encircling the material to be degraded, forming an autophagosome (Parzych and Klionsky, 2014). As the autophagosome forms, microtubule-associated protein 1A/1B-light chain 3, LC3, is converted from its ubiquitous cytosolic form, LC3-I, to LC3-II, which binds to the forming autophagosome membrane, increasing its stability (Bauckman et al., 2015). Upon completion, the autophagosome fuses with a lysosome to form an autolysosome, at which point the internal material is degraded. LC3-II is then recycled back to LC3-1 or degraded.

Using LC3B-II accumulation and P62 degradation as indicators of autophagic flux (Klionsky et al., 2021) we show that antibiotics with anti-*Wolbachia* activity increase autophagy flux in C6/36 and SF9 cell lines and *Brugia malayi*, but not in mammalian cells, and that this activity is blocked by the inhibition of autophagy. This effect is also seen in *Wolbachia-*free cells and is not preceded by ROS production. Furthermore, *Wolbachia* purified from C6/36 cells and treated with anti-*Wolbachia* drugs showed no change in viability or ability to reinfect cells. Collectively, our results show an essential role of host autophagy induction for the efficacy of anti-*Wolbachia* drugs.

## Materials and methods

### Cell culture and *B. malayi* maintenance

The *Aedes albopictus* C6/36 cell line were originally purchased from European Collection of Authenticated Cell Cultures (ECACC) (clone C6/36, 89051705). These cells were stably infected with *Wolbachia pipientis w*AlbB strain derived from Aa23 cells as previously described and are herein referred to as C6/36*Wp* (O’Neill et al., 1997; Turner et al., 2006). Both C6/36 and C6/36*Wp* were maintained in Leibovitz media (Gibco, Thermo Fisher Scientific) supplemented with 20% fetal bovine serum (Thermo Fisher Scientific), 2% tryptose phosphate broth (Sigma-Aldrich), and 1% non-essential amino acids (Sigma-Aldrich). Cells were incubated at 26 °C and subpassaged every 7 days using a 1-in-4 dilution.

Spodoptera frugiperda SF9 cells (Gibco) were maintained in SF-900 II media (Gibco, Thermo Fisher Scientific) supplemented with 20% FBS, 2% tryptose phosphate broth and 1% non-essential amino acids. Cells were incubated at 26 °C and subpassaged every 7 days using a 1-in-4 dilution.

Human monocytic leukemia THP-1 cells were maintained in RPMI 1640 media supplemented with 2 mM L-glutamine (Gibco, Thermo Fisher Scientific), 10% FBS, 5% penicillin–streptomycin (Gibco, Thermo Fisher Scientific), and amphotericin B (Gibco, Thermo Fisher Scientific). Cells were cultured in suspension at 37 °C with 5% CO_2_ and subpassaged twice a week using a 1-in-3 dilution.

Madine-Darby canine kidney (MDCK) epithelial cells were maintained in Eagle’s Minimum Essential Medium (EMEM) media (Sigma Aldrich) supplemented with 10% FBS, 5% penicillin–streptomycin and amphotericin B. Cells were cultured at 37 °C with 5% CO_2_ and subpassaged using a 1-in-4 dilution when a confluency of approximately 80% was reached. Cells were detached using 0.25% Trypsin–EDTA (Gibco, Thermo Fisher Scientific).

The *B. malayi* life cycle is maintained at Liverpool School of Tropical Medicine (LSTM) and were originally obtained from TRS laboratories. Microfilariae (mf) were extracted from the peritoneal cavity of gerbils as previously described (Griffiths et al., 2010). Mf were cleaned and filtered in RPMI 1640 culture media (Gibco, Thermo Fisher Scientific) using PD-10 desalting columns (GE Healthcare) supplemented with 2 mM L-glutamine, 25 mM Hepes, 100 U/mL penicillin–streptomycin (Thermo Fisher Scientific) and 2.5 mg/mL amphotericin B (Thermo Fisher Scientific) at 37 °C. Adult female worms were prepared in the same media.

### Chemical compounds

The antibiotics used in this study (doxycycline, rifampicin, moxifloxacin, sparfloxacin, levofloxacin, ciprofloxacin, amoxicillin, streptomycin, minocycline) were purchased from Sigma Aldrich. All were solubilized in DMSO to a stock concentration of 10 mM. Rapamycin (2.47 mM) and wortmannin (10 mM) were purchased as a ready-made solution (Sigma-Aldrich).

### Immunofluorescence assay (IFA)

Cells were seeded at a density of 50,000 cells/mL in 2 mL of L-15 media on coverslips inside shell vial tubes (Thermo Fisher Scientific). Cells were incubated overnight to allow adherence before the treatment with eight different antibiotics (doxycycline, rifampicin, moxifloxacin, sparfloxacin, levofloxacin, ciprofloxacin, amoxicillin, and streptomycin) or DMSO as a control. Rapamycin, which induces autophagy through the inhibition of mTORC1, and wortmannin, which inhibits autophagy through inhibition of phosphatidylinositol-3-kinase (PI3K), were used as controls (Powis et al., 1994; Voronin et al., 2012; Yang et al., 2013). After the duration of the treatment, the cells were fixed and permeabilised for 30 min using 4% formaldehyde (Thermo Fisher Scientific) in PBS containing Triton x100 (PBS-T). Cells were washed and blocked with 1% bovine serum albumin (BSA) (Sigma Aldrich) for 15 min. The primary antibodies, rabbit anti-LC3B (Invitrogen) and rabbit anti-p62 (Cell Signaling) were diluted in blocking buffer at a 1:400 dilution and cells were incubated overnight at 4 °C. The secondary antibodies goat anti-rabbit fluorescein isothiocyanate Ds grate (FITC) (Invitrogen) and goat anti-rabbit Tetramethylrhodamine (TRITC) (Sigma Aldrich) were used to target LC3B-II and P62, respectively, and were added to cells for 1 h at room temperature. Coverslips were mounted on microscope slides with diamidino-2-phenylindole (DAPI) mounting medium (Vectashield). Images were taken using a Zeiss LSM 880 confocal scanning microscope at 63x magnification using Z-stack 3D images. Three images per slide were taken and used in the analysis.

### Quantification of autophagy induction in microfilariae and cell lines through western blot analysis

*Brugia malayi* mf and SF9, THP-1 and MDCK cells were tested for autophagy induction in response to the antibiotics by western blot. LC3B and p62 were again used to monitor autophagy induction. During autophagy, LC3BI is converted to LC3BII which is seen at a different molecular weight on the immunoblot. As in the IFA, depletion of p62 is an indication of autophagy.

Cells were cultured at a density of 1 × 10^6^ cells per 5 mL in T-25 flasks (Thermo Fisher Scientific) for 3 days in media containing the antibiotics at 5 μM or DMSO control. For the time point assay, C6/36 cells were incubated with the same antibiotics for 0, 1, 3, 5, and 7 days. Cells were then lysed with RIPA lysis buffer (Thermo Fisher Scientific) with added protease inhibitor mix (GE Healthcare) at 10 μL/1 mL for 5 min. *B. malayi* mf were cultured at a density of 10,000 mf/well in a flat-bottom 96-well plate for 3 days in media containing the antibiotics. Four wells were pooled to yield appropriate protein concentrations and were then lysed with 50 μL Tissue Extraction Reagent (Invitrogen, Thermo Fisher Scientific). Tissue lysates were homogenized using a Pellet pestle motor (Kimble) and incubated for 5 min.

Lysates were reduced using NuPAGE reducing agent (Thermo Fisher Scientific) and separated by SDS-PAGE using NuPAGE 6–12% Bis Tris Bolt plus polyacrylamide gel (Thermo Fisher Scientific) at a voltage of 130 v for 1 h with NuPAGE MES running buffer (Thermo Fisher Scientific) containing NuPAGE antioxidant reagent (Thermo Fisher Scientific). Proteins were then transferred into a nitrocellulose membrane with a pore size of 0.22 μM (Amersham, GE Healthcare). After blocking with 5% BSA, the membranes were probed with the appropriate antibodies (Table 1). SuperSignal Chemilluminscent substrate HRP system (Peirce, Thermo Fisher Scientific), was added to the membranes for 5 min, and then exposed to x-ray CL Xposure films (Thermo Fisher Scientific) and imaged using Photons Developer Instrument.

**Table 1:** Primary and secondary antibodies used in western blot analysis, along with their target, species, dilution, and type of blocking buffer.

Primary antibody					Secondary antibody				
Target	Species	Dilution	Blocking buffer	Supplier	Target	Species	Dilution	Blocking buffer	Supplier
LC3B	Rabbit	1:1000	5% non-fat milk in TBS-T	Novus	Rabbit	Goat	1:5000	5% non-fat milk in TBS-T	Cell signaling
P62	Rabbit	1:1000	5% BSA in TBS-T	Cell signaling	Rabbit	Monkey	1:10000	5% BSA in TBS-T	GE Healthcare
Beta actin	Mouse	1:1000	5% BSA in TBS-T	Cell signaling	Mouse	Rabbit	1:20000	5% BSA in TBS-T	Sigma-Aldrich

### Dose response and time-point experiments

C6/36 and C6/36*Wp* cells were seeded at 10,000 cells/mL in a 96-well plate. For the dose–response assay, cells were exposed to doxycycline between 0.125–10 μM, and rifampicin, moxifloxacin, sparfloxacin, levofloxacin, ciprofloxacin, amoxicillin and streptomycin between 0.125–20 μM for 3 days and DMSO as a control. For the time-point assay, cells were treated with the same antibiotics at 5 μM for 0, 1, 3, 5, and 7 days. For the autophagy inhibition assay, cells were treated with doxycycline (5 μM), rifampicin (5 μM), wortmannin (10 μM), l-asparagine (10 mM) or a combination for 7 or 14 days.

Genomic DNA was extracted from each sample using the QIAmp mini-DNA kit (Qiagen). A quantitative polymerase chain reaction (qPCR) was performed using primers against the 16 s rRNA *Wolbachia* gene, normalized to *Aedes albopictus* 18 s rRNA gene (Integrated DNA Technologies) (Table 2). Gene copy numbers were normalized and expressed as a 16 s:18 s ratio.

**Table 2:** Complete sequence list of qPCR primer pairs used.

Primers	Sequence
*16S* F	5′-TTGCTATTAGATGAGCCTATATTAG-3′
*16S* R	5′-GTGTGGCTGATCATCCTCT-3′
*18S* F	5′-CCGTGATGCCCTTAGATGTT-3′
*18S* R	5′-ATGCGCATTTAAGCGATTTC-3′
*wBMWSP* F	5′ CCC TGC AAA GGC ACA AGT TAT TG 3′
*wBMWSP* R	5′ CGA GCT CCA GCA AAG AGT TTA ATT 3′
*BMGST* 1368	5′ GAG ACA TCT TGC TCG CAA AC 3′
*BMGST* 1632	5′ ATC ACG GAC GCC TTC ACA G 3′

### Efficacy of anti-*Wolbachia* compounds in combination with autophagy inhibitors, in *B. malayi*

Live *B. malayi* mf and adults were treated with doxycycline (5 μM), rifampicin (5 μM), wortmannin (10 μM), l-asparagine (10 mM) or a combination, for 6 days. Five and eight biological replicates were performed for mf and female adult worms, respectively.

Genomic DNA was extracted from whole body samples using the QIAmp mini-DNA kit (Qiagen). For adult worms only, prior to overnight incubation, samples were also incubated for 30 min at 56 °C and then vortexed. DNA was then amplified using *Wolbachia* Surface Protein (w*Bm wsp*) and glutathione *S*-transferase (*Bm gst*) genes, and gene copy number quantified by qPCR as previously described (Table 2) (McGarry et al., 2004).

RNA was extracted using MiRCURY RNA Isolation kit (Exiqon), as per the manufacturer’s instructions. The RNA concentration and purity was assessed using a Nanodrop 1,000 (Thermo Fisher Scientific). Approximately 0.25 μg of purified RNA were then used a in reverse transcriptase polymerase chain reaction (RT-PCR) to synthesize complementary DNA (cDNA) using Superscript III RT (Invitrogen, Thermo Fisher scientific). Primers targeting *wBmWSP* and *BmGST* were quantified using qPCR (Table 2). These forward and reverse primers were diluted at a final concentration of 0.5 μM. 1.2 μL of each primer was added to 10 μL SYBR green mix with 1 μL of amplified cDNA and 0.4 μL MgCl_2_ diluted in RNAse-free water (Thermo Fisher Scientific) to provide a total volume of 20 μL in each reaction well. The PCR cycles were set as follows: at 95 °C for 15 min, 40 cycles at 94 °C for 30 s, 62 °C for 30 s and at last 72 °C for 1 min per kb. The ratio of *wBMWSP* to *BmGST* was then calculated.

### Measurement of intracellular ROS

C6/36 and C6/*36Wp* cells were seeded at 20,000 cells per well a 96 well plate and incubated overnight until >80% confluent. To measure ROS generation in response to the drugs, cells were incubated in 10 μM CM-H_2_DCFDA (Invitrogen) for 30 min in the dark at 26 °C, after which the drugs were added. Pyocyanin (200 mM), a known inducer of ROS, was used as a positive control. Fluorescence was read on a kinetic read at an excitation/emission of 500/527 on a Varioskan plate reader, every hour for 24 h.

### Viability of drug treated extracellular *Wolbachia*

*Wolbachia* were purified from C6/36*Wp* cells as previously described (Rasgon et al., 2006). Briefly, confluent cells were detached by scraping and collected in a tube. Cells were centrifuged at 400 x *g* for 5 min to pellet, the supernatant media removed and the pellet resuspended in 10 mL fresh L-15 media. The suspension was then vortexed for 5 min with approximately 100 sterile borosilicate glass beads to lyse cells. The lysate was centrifuged to remove cellular debris, and the supernatant filtered through a 5 μm syringe filter. The filtrate was centrifuged at 18,400 x *g* at 4 °C for 5 min on a 250 mM sucrose cushion to pellet *Wolbachia*. The pellet was resuspended in L-15 media and filtered through a 2.74 μm syringe filter to remove residual cellular debris. For re-infection experiments, the filtrate was passed again through a 0.45 μm syringe filter. The bacterial density was adjusted to an OD_600_ of approximately 0.08. Hundred microliter of *Wolbachia* was added per well to a 96 well-plate directly after 2.74 μm filtration to avoid clumping. This was made up to 200 μL with 2X drug in L-15 media and incubated for 7 days at 26 °C. All antibiotics were used at 5 μM final concentration, and AWZ1066S at both 50 nM and 5 μM.

Viability of the *Wolbachia* was determined by LIVE/DEAD™ *Bac*Light™ Bacterial viability kit (Invitrogen). For killed controls, media was replaced with 70% isopropyl alcohol and incubated at room temperature for 1 h. All wells were washed once with 0.85% NaCl. *Bac*Light stain was made up (1:1 component A to component B) in 0.85% NaCl, as per the manufacturer’s instructions. The buffer was replaced with the stain and incubated for 15 min in the dark. Images were taken using a Zeiss LSM 880 confocal scanning microscope at 63x magnification. There were three wells per treatment, three images were taken per well, and a total of three independent experiments were done, all of which were used in the analysis. The percentage of live bacteria was determined through ImageJ. First, the images were converted to gray-scale, then the threshold for fluorescence was set based on the positive and negative control images. This threshold was then kept the same for all images of each experiment. The area taken up by each fluorescence channel was then quantified and the percentage live determined by dividing the area taken up by the green (live) channel by the total area of red (dead) and green (live) channels.

BacLight stain viability is based upon dead bacteria having a compromised membrane. To ensure that the *Wolbachia* were viable, and not dead with an intact membrane, drug-treated extracellular *Wolbachia* were then incubated with C6/36 cells to assess their ability to reinfect. For re-infection studies, *Wolbachia* were purified and drug treated as above. After 7 days, the bacteria pellets were washed 3 times in L-15 media, resuspended in L-15 media, and added to confluent C6/36 cells, seeded in a 96-well plate. The plate was centrifuged at 2,600 x *g* for 1 h then incubated at 26 °C for 7 days. Cells were stained with Hoechst 33342 (Thermo Fisher) and SYTO ™-11 (Thermo Fisher) to visualize cell nuclei and *Wolbachia*, respectively. Images were taken using a Zeiss LSM 880 confocal scanning microscope at 63x magnification. There were three wells per treatment, three images taken per well, and a total of two independent experiments done. The pinhole and laser strength settings were kept the same throughout each experiment. The number of *Wolbachia*-infected cells were then quantified as a percentage of total cells per image.

### Data analysis

All data analysis was done in R (v4.4.2). All plots were made using ggplot2 (Wickham and Sievert, 2009). Data was tested for normality using the Shapiro–Wilk Normality Test (Royston, 1982). An ANOVA or Kruskal–Wallis Rank Sum Test was used to determine any differences between groups and if so, either Tukey’s honest significance test or Conover-Iman Test of multiple comparisons using rank sums was used (Kruskal and Wallis, 1952; Conover and Iman, 1979; Yandell, 2017) with Bonferroni adjustment for multiple testing (Dunn, 1961). Where boxplots are used, the lower and upper hinges correspond to the first and third quartiles and the whiskers extend to the largest value within 1.5× the interquartile range. Data points outside of this reach are defined as outlying and are plotted as individual points.

## Results

### Anti-*Wolbachia* drugs induce autophagy in *B. malayi* and C6/36 cells as well as *Wolbachia*-free insect cells

**Figure 1:**
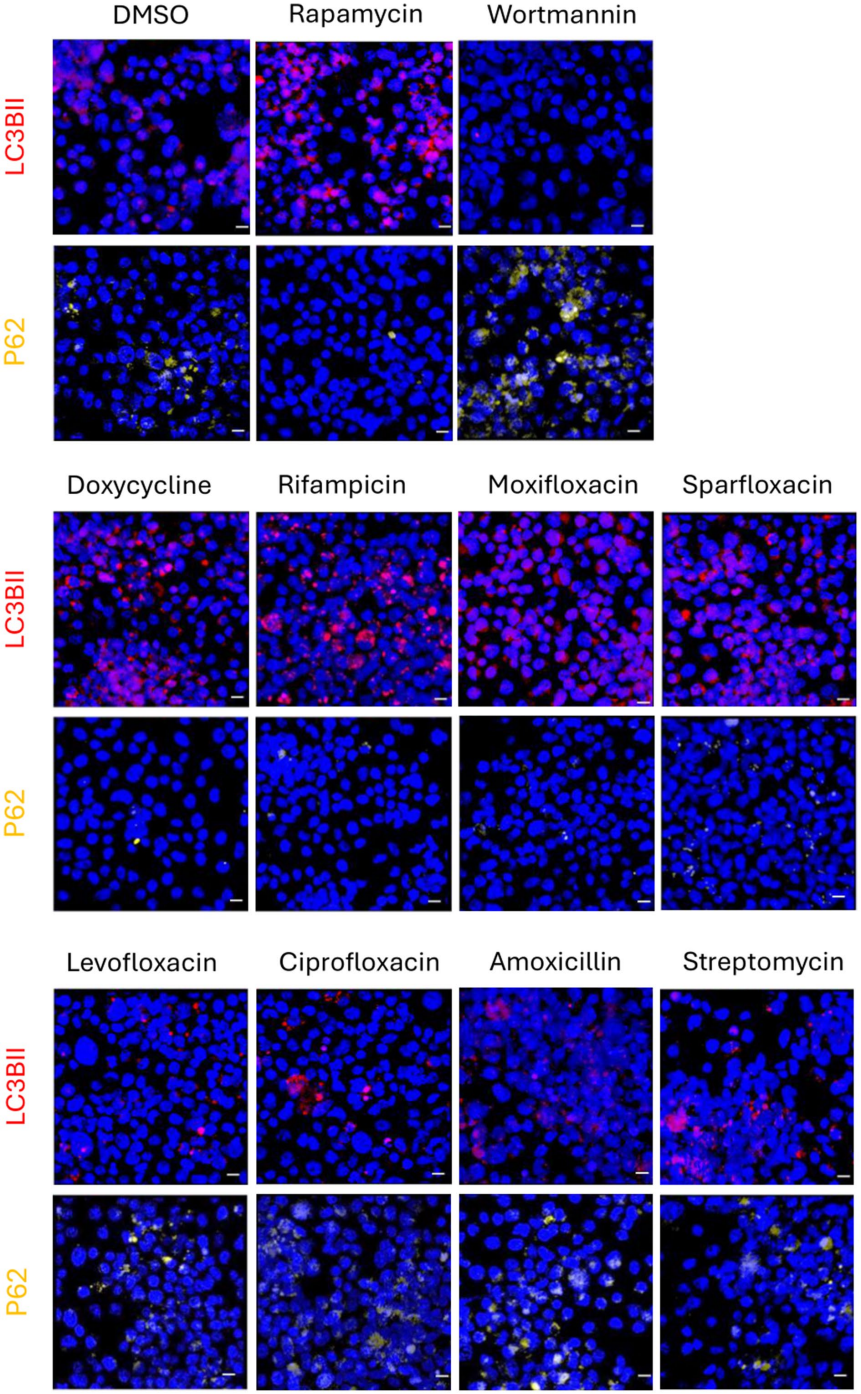
Immunofluorescence staining in C6/36Wp cells for different antibiotics using autophagic markers for LC3B-II and p62. C6/36 cells infected with wAlbB were treated with DMSO (vehicle control representing basal autophagy), rapamycin at 5 μM (positive control), wortmannin at 10 μM (negative control), four anti-*Wolbachia* antibiotics: doxycycline, rifampicin, moxifloxacin and sparfloxacin, and four non-anti-*Wolbachia* antibiotics: levofloxacin, ciprofloxacin, amoxicillin and streptomycin, all at 5 μM for 3 days. Cells were fixed, permeabilised and incubated with autophagy primary and secondary antibodies. DAPI was used to stain cell nuclei (blue fluorescence). Autophagy activation is shown by an increase in red puncta (increase in LC3B-II due to autophagosomes formation) and by a decrease in yellow puncta (decrease in p62 due to autophagic degradation). Autophagy flux is seen in response to anti-*Wolbachia* antibiotics but not to those without anti-*Wolbachia* activity.

**Figure 2:**
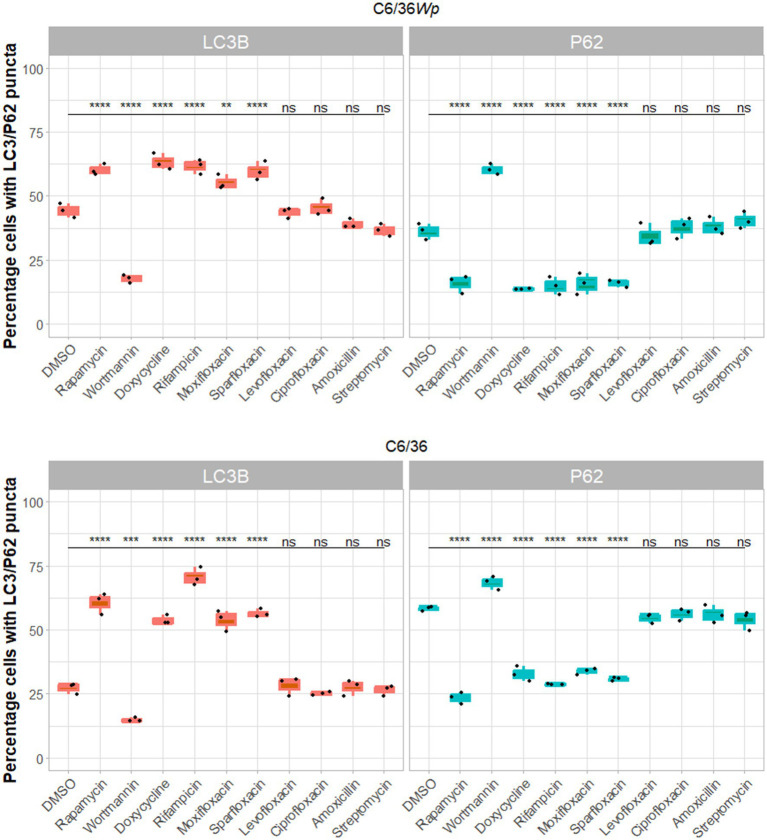
*Wolbachia* infected **(A)** or *Wolbachia*-free C6/36 cells **(B)** were treated with DMSO (vehicle control representing basal autophagy), rapamycin at 5 μM (positive control), wortmannin at 10 μM (negative control), four anti-*Wolbachia* drugs: doxycycline, rifampicin, moxifloxacin and sparfloxacin, and four non-anti-*Wolbachia* drugs: levofloxacin, ciprofloxacin, amoxicillin and streptomycin, all at 5 μM for 3 days. Cells were fixed, permeabilised and incubated with autophagy marker antibodies. Autophagy flux activation is shown by an increase in LC3BII puncta and a decrease in p62 puncta. Data was analyzed and plotted using R. Significance compared to DMSO was tested using ANOVA and Tukey’s honest significance test. Autophagy flux is seen in response to anti-*Wolbachia* antibiotics but not to those without anti-*Wolbachia* activity, in both cell lines.

We examined autophagic induction by eight antibiotics using immunofluorescence staining assay in C6/36*Wp* (Figures 1, 2A) and *Wolbachia*-free C6/36 cells (Figure 2B). Percentage of cells with positive LC3B and P62 puncta were quantified, where an increase in LC3B and a decrease in P62 is indicative of autophagy activation. Treatment with rapamycin, a known inducer of autophagy, led to an increase in LC3B puncta and a decrease in P62, whereas treatment with wortmannin, an inhibitor of early-stage autophagy, shows the inverse pattern (Figure 1).

All four anti-*Wolbachia* antibiotics (doxycycline, rifampicin, moxifloxacin, and sparfloxacin) showed activation of autophagy in C6/36*Wp* cells, through an increase in LC3B positive cells and a decrease in P62 positive cells (Figure 2A). This was similar to that of the rapamycin treatment. Conversely, the remaining antibiotics without anti-*Wolbachia* activity (levofloxacin, ciprofloxacin, amoxicillin, and streptomycin) did not exhibit any observable difference in LC3B and P62 positive cells compared to the DMSO control. This was also seen in *B. malayi* mf (Supplementary Figure S1A). While conversion of LC3B-I to LC3B-II was seen, this was not accompanied by degradation of P62. This does not, therefore, suggest an increase in autophagy flux, and could be due to an accumulation of autophagosome membranes or a block in the later stages of autophagy (Klionsky et al., 2021).

Interestingly, the same pattern was shown in *Wolbachia*-free C6/36 cells and SF-9 cells (Figure 2B and Supplementary Figure S1B). One notable difference between the infected and uninfected C6/36 cells is the increased induction of autophagy in the DMSO control of C6/36*Wp* cells compared to *Wolbachia* free cells.

No increase in autophagy flux was seen in response to any of the antibiotics (doxycycline, rifampicin, moxifloxacin, sparfloxacin, levofloxacin, ciprofloxacin, amoxicillin, streptomycin) tested in either mammalian cell line (Supplementary Figures S1C,D).

Autophagy was also induced in C6/36 and C6/36*Wp* cells in response to novel anti-*Wolbachia* drugs, TylAMac, fusidic acid, and AWZ1066S (Supplementary Figure S2). Novel anti-*Wolbachia* drugs here refers to drugs, which have either been specifically designed to target *Wolbachia* or new repurposed drugs that are active against *Wolbachia.*

### Dose-dependency of efficacy correlates with autophagy induction

To evaluate whether effective antibiotic concentrations against *Wolbachia* also activate autophagy, and whether a dose-dependent effect could be seen, we measured each drug’s anti-*Wolbachia* activity alongside autophagy induction.

**Figure 3:**
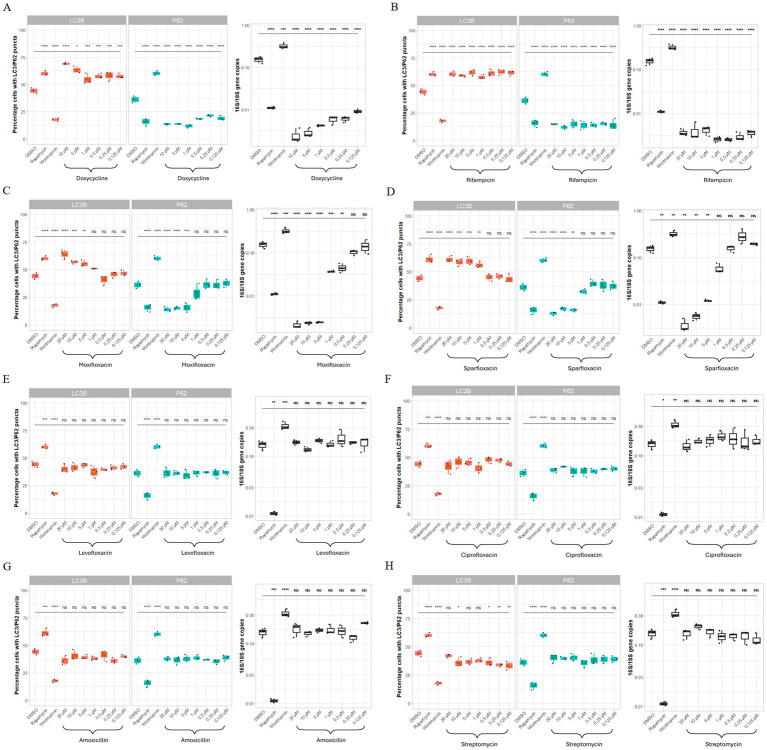
Concentration-dependency of antibiotic induced autophagy activation by anti-*Wolbachia* drugs; doxycycline, rifampicin, moxifloxacin and sparfloxacin, corresponds to drug efficacy in *Wolbachia*-infected C6/36 cells (W+). C6/36 cells infected with wAlbB were treated with DMSO (vehicle control representing basal autophagy), rapamycin at 5 μM (positive control), wortmannin at 10 μM (negative control) and either drugs with anti-Wolbachia activity (doxycycline **[A]**, rifampicin **[B]**, moxifloxacin **[C]**, sparfloxacin **[D]**) or without anti-Wolbachia activity (levofloxacin **[E]**, ciprofloxacin **[F]**, amoxicillin **[G]**, streptomycin **[H]**) for 3 days. For immunofluorescence, cells were fixed, permeabilised and incubated with autophagy marker antibodies. For autophagy activation, autophagic markers: LC3B-II and p62 expression increased and decreased, respectively. Boxplots represent % cells with LC3B-II or p62 positive puncta for each treatment group with three different sections were imaged, where each section contained ≥50 cells. In the same treatment groups, qPCR was used to quantify *Wolbachia* DNA extracted from cells were amplified using *Wolbachia 16s* rRNA gene normalized to *Aedes albopictus 18s* rRNA gene. Graphs represent *16s:18s* gene copies (log10) in three biological repeats per treatment group. Data was analyzed and plotted using R. Significance compared to DMSO was tested using ANOVA and Tukey’s honest significance test. Only concentrations and compounds with activity against *Wolbachia* are seen to induce autophagic flux.

Both doxycycline and rifampicin induced autophagy and significantly reduced *Wolbachia* load at all tested concentrations (Figures 3A,B). The two anti-*Wolbachia* fluoroquinolones, moxifloxacin and sparfloxacin, induced autophagy at the higher concentrations only (between 5 and 20 μM) corresponding with a significantly lower *Wolbachia* load (>90%) (Figures 3C,D).

No induction of autophagy was seen at any concentration of levofloxacin, ciprofloxacin, amoxicillin or streptomycin and as expected no change in *Wolbachia* load was seen (Figures 3E–H).

Inducing autophagy using rapamycin reduced *Wolbachia* load significantly compared to the DMSO control, whereas inhibition of autophagy using wortmannin, significantly increased *Wolbachia* load (Figure 3). This effect was also observed in the confocal images of C6/36*Wp*.

**Figure 4:**
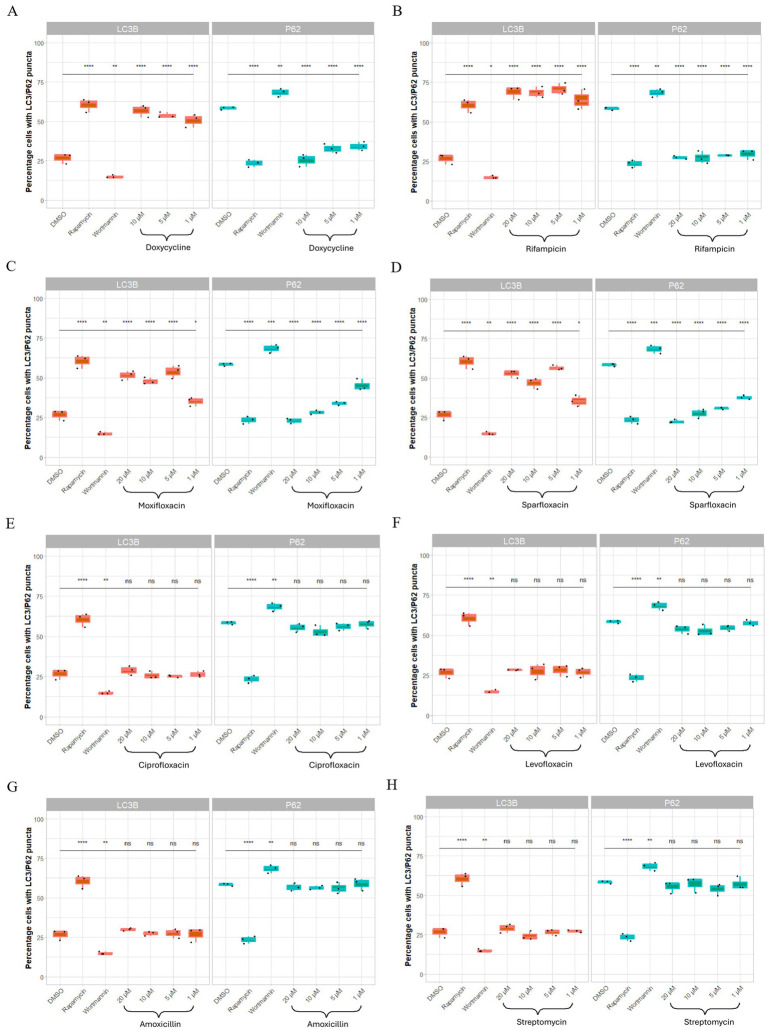
Concentration-dependency of antibiotic induced autophagy activation occurs in uninfected C6/36 cells (W-). C6/36 cells infected with *wAlbB* were treated with DMSO (vehicle control representing basal autophagy), rapamycin at 5 μM (positive control), wortmannin at 10 μM (negative control), anti-*Wolbachia* drugs: doxycycline, rifampicin, moxifloxacin and sparfloxacin **(A–D)** or non-anti-*Wolbachia* drugs; levofloxacin, ciprofloxacin, amoxicillin and streptomycin **(E–H)** at 1–20 μM for 3 days. For immunofluorescence, cells were fixed, permeabilised and incubated with autophagy marker antibodies. For autophagy activation, autophagic markers: LC3B-II and p62 expression increased and decreased, respectively. Boxplots represent % cells with LC3B-II or p62 positive puncta for each treatment group with three different sections were imaged, where each section contained ≥50 cells. Data was analyzed and plotted using R. Significance compared to DMSO was tested using ANOVA and Tukey’s honest significance test. Only concentrations and compounds with activity against *Wolbachia* are seen to induce autophagic flux, even in uninfected cells.

As in C6/36*Wp*, all four anti-*Wolbachia* drugs significantly induced autophagy in C6/36 cells, as expressed by both autophagy markers (Figure 4). The same dose-dependent pattern by moxifloxacin and sparfloxacin was also observed in the absence of *Wolbachia* (Figures 4C,D). Of the antibiotics with no anti-*Wolbachia* activity, again, no induction of autophagy was seen at any concentration tested (Figures 4E–H).

All antibiotics were then tested at different treatment periods in C6/36 cells (Supplementary Figure S3). All four tested anti-*Wolbachia* drugs showed evidence of autophagy induction in all periods, shortest being 1 day. All antibiotics without anti-*Wolbachia* activity failed to induce autophagy at any time point, when measured by both LC3B conversion and P62 degradation (Supplementary Figure S3). Ciprofloxacin and levofloxacin showed some LC3B conversion in the first day of treatment, however this was not accompanied by P62 degradation or maintained in subsequent treatment days (Supplementary Figure S3).

### Inhibition of autophagy blocks efficacy of anti-*Wolbachia* drugs

Next, we explored whether inhibition of autophagy would affect the efficacy of anti-*Wolbachia* drugs, using wortmannin and L-asparagine.

**Figure 5:**
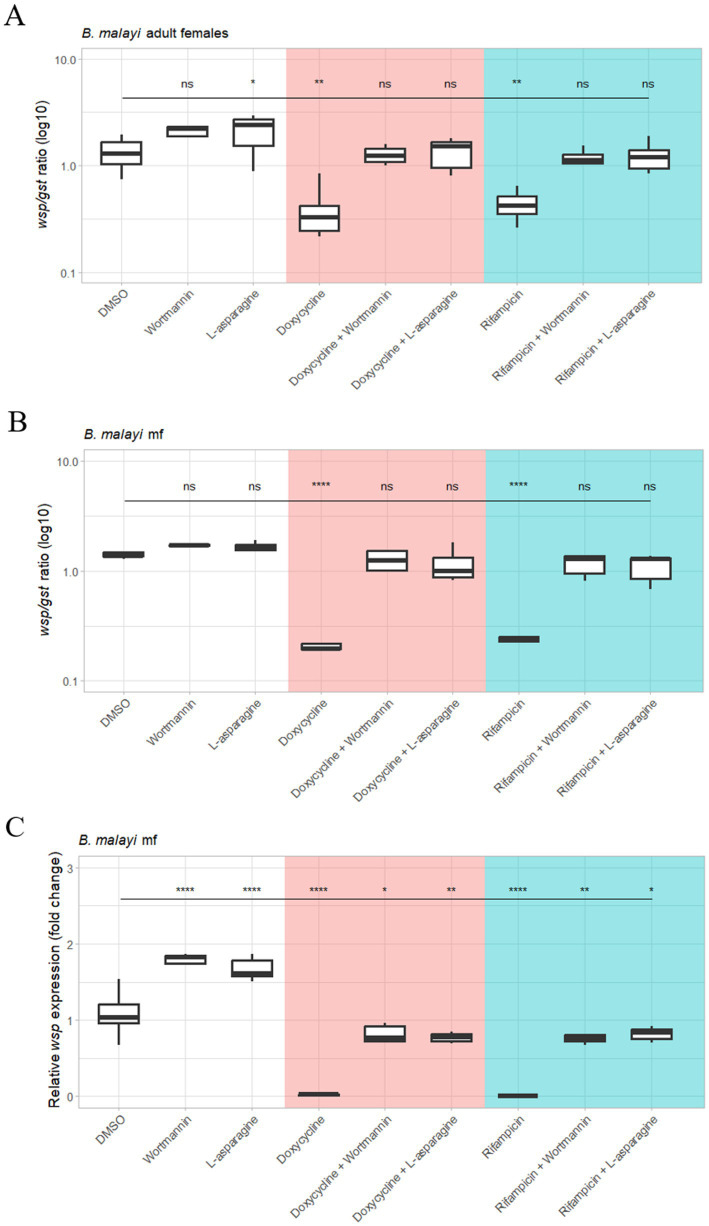
qPCR analysis of *Wolbachia* load and RNA gene expression following autophagy inhibition during drug exposure in *B. malayi* mf and female adult worms. *Brugia malayi* female worms **(A)** and mf **(B)** were treated with doxycycline or rifampicin at 5 μM alone or combined with wortmannin (at 10 μM) or l-asparagine (at 10 mM) for 6 days. In addition, treatment groups containing only wortmannin or l-asparagine were included. DMSO was used as vehicle control. DNA extracted from worms were amplified using *Wolbachia wsp* gene normalized to the nematode *gst* gene. Boxplots represent *wsp:gst* gene copies (log10) in five and eight biological repeats per treatment group, for mf and female worms, respectively. **(C)** *Wolbachia* RNA gene expression following autophagy inhibition during drug exposure in *B. malayi* mf. The relative change in *B. malayi Wolbachia wsp* gene expression levels between untreated mf and different mf groups exposed to the following treatment for 6 days: doxycycline (or rifampicin) at 5 μM alone or combined with wortmannin (at 10 μM) or l-asparagine (at 10 mM). In addition, treatment groups containing only wortmannin or l-asparagine were included. Boxplots represent fold change of gene expression in six biological repeats per treatment group. Data was analyzed and plotted using R. Significance compared to DMSO was tested using ANOVA and Tukey’s honest significance test. Autophagy inhibitors reduce the effect of anti-*Wolbachia* drugs. Statistical significance was at *p* ≤ 0.05. For *p*-value * = 0.01–0.05, ** = 0.01–0.001, *** = 0.001–0.0001, and **** ≤ 0.0001.

In both *B. malayi* mf and adults, doxycycline, and rifampicin significantly reduced *Wolbachia* load, as expected (Figures 5A–C). The addition of autophagy inhibitors wortmannin and L-asparagine to both antibiotic treatments led to *Wolbachia* loads similar to the DMSO control (Figures 5A,B). Treatment with autophagy inhibitors alone also increased *Wolbachia* load significantly, compared to the DMSO control. Apart from the combination of rifampicin and wortmannin in adult female worms, all autophagy inhibitor treatments alone had a significantly higher *Wolbachia* load than their combination with either antibiotic. Since *Wolbachia* DNA may detect both live and dead bacteria in order to determine the viability of *Wolbachia* following these treatments, we also measured RNA expression of *Wolbachia* surface protein (*wsp*) (Figure 5C). A similar pattern was seen, with no *Wolbachia* expression after drug treatment, and comparable to DMSO control with the addition of inhibitors.

### Autophagy induction is not induced by ROS production

**Figure 6:**
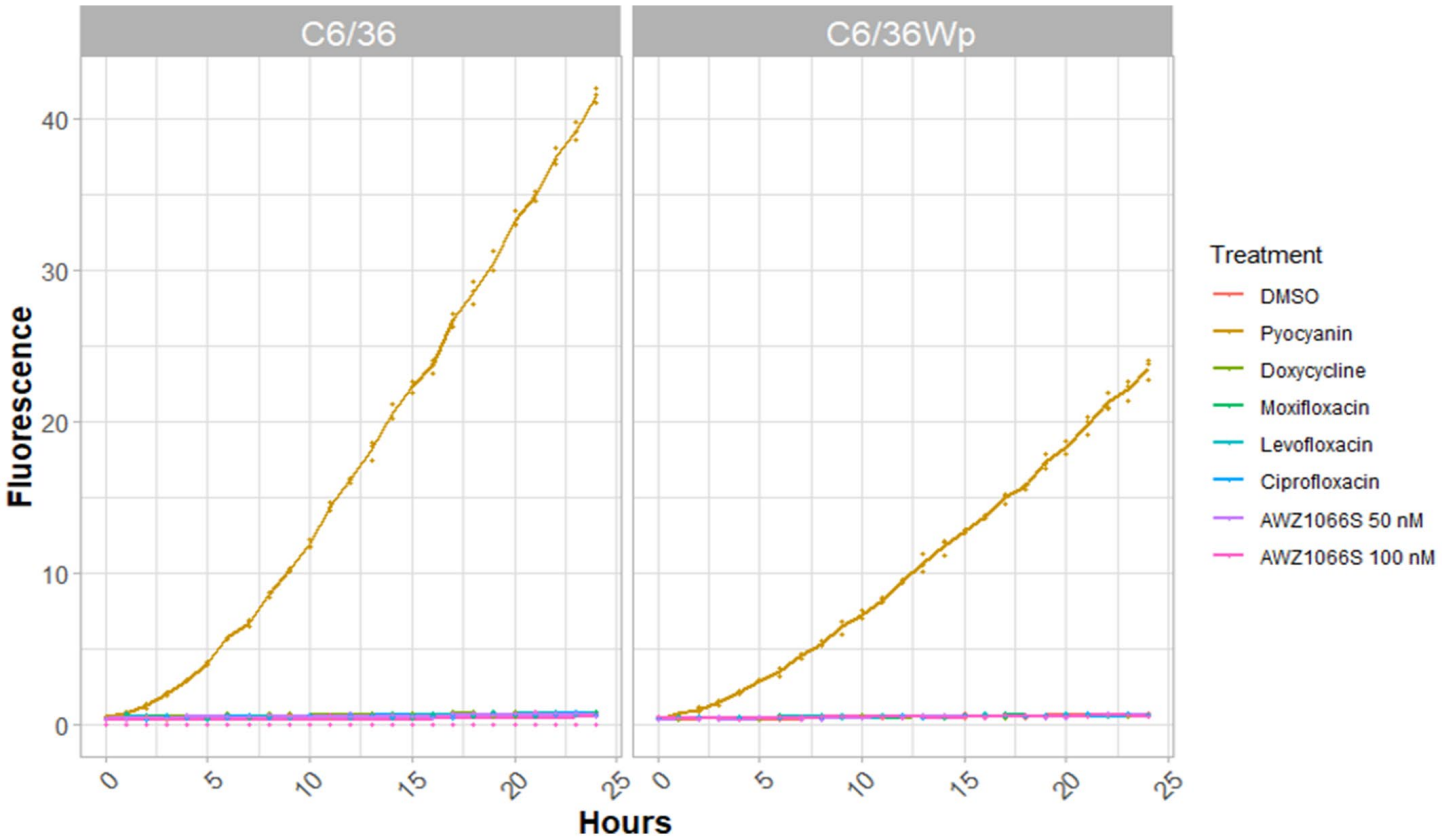
Intracellular ROS production in C6/36 and C6/36*Wp* cells. Cells incubated with CM-H_2_DCFDA dye were treated with anti-*Wolbachia* drugs (doxycycline and moxifloxacin) as well as antibiotics without anti-*Wolbachia* efficacy (levofloxacin and ciprofloxacin) at 5 μM. Cells were also treated with fast-acting anti-*Wolbachia* compound, AWZ1066S, at 50 nM and 100 nM. DMSO and pyocyanin (200 mM) were used as controls. Fluorescence was read every hour for 24 h using a Varioskan plate reader. Data was plotted using R. ROS generation was not seen in response to any anti-*Wolbachia* drug.

Autophagy can be induced by several triggers, including production of ROS. Fluorescence of CM-H_2_DCFDA dye was used as an indicator of the presence of intracellular ROS, and was measured over a period of 24 h, the minimum time shown for autophagy induction to occur (Supplementary Figure S3). No ROS was measured in response to any of the antibiotics in C6/36 cells uninfected or infected with *Wolbachia* (Figure 6). This suggests autophagy is induced independently of ROS generation. Almost double the fluorescence is seen in response to the positive control, pyocyanin, in uninfected cells than *Wolbachia*-infected cells, indicating some level of protection against oxidative stress (Figure 6).

### Extracellular *Wolbachia* viability is unaffected by exposure to anti-*Wolbachia* drugs

**Figure 7:**
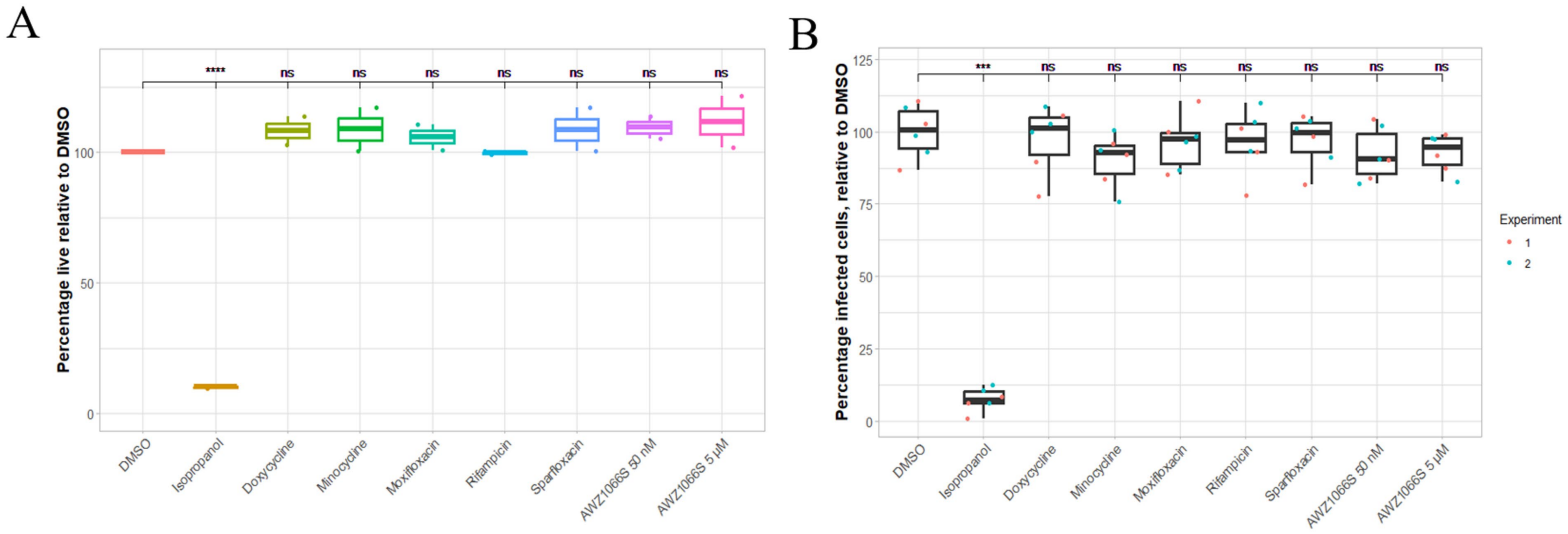
Percentage of live *Wolbachia* after extracellular drug treatment measured by BacLight stain **(A)** and re-infectivity **(B)**. *Wolbachia* purified from C6/36 cells were treated with anti-*Wolbachia* drugs (doxycycline, minocycline, moxifloxacin, rifampicin, sparfloxacin, and AWZ1066S) at 5 μM for 7 days. AWZ1066S was also included at 50 nM. Killed control *Wolbachia* were treated with 70% isopropyl alcohol for 1 h. **(A)** *Wolbachia* were stained with LIVE/DEAD BacLight viability stain and imaged with a confocal microscope. Percentage live bacteria were calculated by area, using ImageJ. **(B)** Treated *Wolbachia* were washed and incubated on C6/36 cells for 7 days. Cells were stained with SYTO-11 and images taken by confocal microscopy. Re-infection rate was measured using ImageJ (Cell Counter plug in). Data was analyzed and plotted using R. Significance compared to DMSO was tested using Kruskal–Wallis test then Conover’s test for multiple comparisons. Extracellular treatment with anti-*Wolbachia* drugs did not affect the viability of *Wolbachia* nor their ability to re-infect C6/36 cells. Statistical significance was at *p* ≤ 0.05. For *p*-value * = 0.01–0.05, ** = 0.01–0.001, *** = 0.001–0.0001, and **** ≤ 0.0001.

To further understand the role of the host cell in anti-*Wolbachia* therapy, *Wolbachia* were isolated from C6/36 cells and treated with anti-*Wolbachia* drugs extracellularly. A range of drugs were used, all at concentrations which deplete intracellular infection, as well as an extremely high dose of AWZ1066S at 2000X the intracellular EC_50_. No treatment had any effect on *Wolbachia* viability after 7 days (Figure 7A and Supplementary Figure S4). BacLight stain viability is determined by the integrity of the cell membrane and so to ensure the *Wolbachia* were not dead with an intact membrane, their ability to re-infect after extracellular drug treatment was assessed. *Wolbachia* were incubated with C6/36 cells for 7 days so to allow for replication and further re-infection, rather than initial infection, to get a more complete picture of viability. All treated bacteria infected cells to a similar level as the DMSO-treated control, aside from the isopropyl treated control group (Figure 7B). Together, this highlights the critical role of the host cell in *Wolbachia* clearance by the anti-*Wolbachia* drugs.

## Discussion

Antibiotics that deplete *Wolbachia* infection effectively block the development of larval filarial nematodes in the mammalian host, and in adult worms leads to sterility and death (Taylor et al., 2005a; Taylor et al., 2013b). Mf may circulate for several months after treatment, but rapidly lose their ability to be transmitted through the mosquito vector (Quek et al., 2022). This has made anti-*Wolbachia* therapy an attractive macrofilaricidal treatment with transmission blocking activity, but the process through which these drugs kill *Wolbachia* is unknown. Here, we show that a range of antibiotics with anti-*Wolbachia* activity induce autophagy in *B. malayi* and C6/36*Wp*, whereas those without anti-*Wolbachia* efficacy did not. Only drug concentrations that effectively depleted *Wolbachia* (>90%) induced autophagy, showing the anti-*Wolbachia* activity of the drugs is closely linked with their ability to induce autophagy. Furthermore, inhibition of either early or late-stage autophagy with wortmannin and L-asparagine, respectively, completely blocked the efficacy of both doxycycline and rifampicin in *B. malayi*. This is further evidence for autophagic flux as the main killing mode of action of anti-*Wolbachia* drugs. This is promising, in terms of resistance, since due to the vital role of autophagy for survival any mutations are likely to be detrimental.

Interestingly, the same pattern of autophagy induction was also seen in uninfected C6/36 cells, as well in SF9 cells, suggesting the mode of action of these drugs is independent of *Wolbachia* infection. Since the known targets of these antibiotics are bacterial, this would suggest an off-target effect in the host cell which leads to the induction of autophagy. Notably, no tested anti-*Wolbachia* drug activated autophagy flux in either mammalian cell line, highlighting their restricted role in insect cells and nematodes (Supplementary Figures S1C,D). This is of importance since activation of autophagy in mammalian cells could lead to adverse effects. This could be due to an invertebrate specific pathway, or invertebrate specific trigger, to autophagy induction. Since the antibiotics are mostly used to target human bacterial infections, the research surrounding them is focused on mammalian systems, so off-target effects in invertebrates are not well studied.

Many intracellular bacteria must subvert or even exploit host autophagy responses to survive (Cemma and Brumell, 2012; Castrejón-Jiménez et al., 2015). Drugs against intracellular bacteria can take advantage of this and induce autophagy as part of their MOA. A common process through which drugs induce autophagy is through the production of ROS, for example, by anti-tuberculosis drugs, isoniazid and pyrazinamide (Kim et al., 2012). Production of ROS due to mitochondrial dysfunction has previously been shown in response to several antibiotics (Esner et al., 2017; Xiao et al., 2019). For this reason, ROS as an inducer of autophagy was investigated. However, no ROS was generated in response to any of the drugs tested within a period of 24 h, the shortest period shown for autophagy induction to take place.

Other drugs against intracellular bacteria can act through ROS-independent autophagy. Anti-*Salmonella* compounds D61 and AR12 do so through PI3K signaling, the latter via inhibition of PDK-1/Akt signaling and endoplasmic reticulum stress (Chiu et al., 2009; Nagy et al., 2019). Endoplasmic reticulum stress can lead to ER-mediated autophagy and since *Wolbachia* is often found interacting with the ER, and a *Wolbachia* infection has been associated with ER stress and triggering of the unfolded protein response in *Ae. aegypti* cells, this could be a plausible mode of action in anti-*Wolbachia* drugs (Geoghegan et al., 2017; Fattouh et al., 2019). Anti-tuberculosis drug, Gefitinib, activates autophagy through the inhibition of epidermal growth factor receptor (EGFR) in macrophages (Stanley et al., 2014). In insects, EGFR is linked to ecdysone biosynthesis, a hormone which is involved in autophagy induction (Rusten et al., 2004; Cruz et al., 2020). These examples induce autophagy in mammalian cells, unlike anti-*Wolbachia* drugs, but many autophagy pathways are well conserved across eukaryotes but may be triggered through different processes.

A recent study has reported that some autophagy-modulating compounds which are reported to induce autophagy in mammalian systems, are effective in reducing *Wolbachia* levels and directly induce damage to nematode tissues in *Brugia pahangi* (Voronin et al., 2024). This suggests these compounds are able to induce autophagy in the human host as well as the nematode. Though this may be effective *in vitro*, compounds which induce autophagy only in the parasite would be less likely to cause off-target effects in humans. Hargitai et al. (2022) investigated the role of autophagy in the clearance of *Wolbachia* in *Drosophila* in response to the anti-*Wolbachia* compound, tetracycline. The treatment led to an increased autophagy-mediated degradation of *Wolbachia*, which was inhibited in autophagy mutant flies. They hypothesized that autophagy was a response to *Wolbachia* damaged by the antibiotic treatment, since starvation-induced autophagy did not affect *Wolbachia* load. However, in our work we saw antibiotic induced autophagic flux even in the absence of *Wolbachia* infection. The pathway through which autophagy is induced by anti-*Wolbachia* compounds could be different to that induced by starvation.

To see if anti-*Wolbachia* drugs had any effect on extracellular *Wolbachia*, we assessed their viability both through BacLight membrane stain and through their ability to re-infect C6/36 cells after drug treatment. In both assays, drug-treated extracellular *Wolbachia* were still viable, suggesting there is no direct killing action. Since *Wolbachia* are obligately intracellular, with a degenerate genome, it could be that they are metabolically quiescent when extracellular and are therefore unaffected by anti-*Wolbachia* drugs, many of which are known to target protein synthesis (Foster et al., 2005). Many species of bacteria can enter a dormant stage, associated with drug resistance, during which metabolism and replication decreases significantly (Ayrapetyan et al., 2015). *Staphylococcus aureus* drug-resistant bacteria are able to persist intracellularly, with a low growth rate but still metabolically active (Peyrusson et al., 2020). At this point, they are transcriptionally different from active bacteria, showing an increase in gene expression related to the stress response. Further work is underway to assess the transcriptional activity of *Wolbachia* extracellularly.

In conclusion, we have shown that autophagy is required for the activity of anti-*Wolbachia* drugs, and that this is only induced in invertebrate cells. This process is independent of *Wolbachia* infection and does not act through the production of ROS. We have also shown the drugs do not directly kill extracellular *Wolbachia*, further highlighting the critical role of the host cell in anti-*Wolbachia* efficacy.

## Data Availability

The raw data supporting the conclusions of this article will be made available by the authors, without undue reservation.

## References

[ref1] Aljayyoussi G. Tyrer H. E. Ford L. Sjoberg H. Pionnier N. Waterhouse D. . (2017). Short-course, high-dose rifampicin achieves *Wolbachia* depletion predictive of curative outcomes in preclinical models of lymphatic filariasis and onchocerciasis. Sci. Rep. 7, 1–11. doi: 10.1038/s41598-017-00322-5 28303006 PMC5428297

[ref2] Ayrapetyan M. Williams T. C. Oliver J. D. (2015). Bridging the gap between viable but non-culturable and antibiotic persistent bacteria. Trends Microbiol. 23, 7–13. doi: 10.1016/j.tim.2014.09.004, 25449050

[ref3] Bauckman K. A. Owusu-Boaitey N. Mysorekar I. U. (2015). Selective autophagy: Xenophagy. Methods 75, 120–127. doi: 10.1016/j.ymeth.2014.12.005, 25497060 PMC4355331

[ref4] Castrejón-Jiménez N. S. Leyva-Paredes K. Hernández-González J. C. Luna-Herrera J. García-Pérez B. E. (2015). The role of autophagy in bacterial infections. Biosci. Trends 9, 149–159. doi: 10.5582/bst.2015.01035, 26166368

[ref5] Cemma M. Brumell J. H. (2012). Interactions of pathogenic bacteria with autophagy systems. Curr. Biol. 22, R540–R545. doi: 10.1016/j.cub.2012.06.001, 22790007

[ref6] Chiu H.-C. Kulp S. K. Soni S. Wang D. Gunn J. S. Schlesinger L. S. . (2009). Eradication of intracellular *Salmonellaenterica* serovar typhimurium with a small-molecule, host cell-directed agent. Antimicrob. Agents Chemother. 53, 5236–5244. doi: 10.1128/aac.00555-09 19805568 PMC2786354

[ref7] Clare R. H. Bardelle C. Harper P. Hong W. D. Börjesson U. Johnston K. L. . (2019). Industrial scale high-throughput screening delivers multiple fast acting macrofilaricides. Nat. Commun. 10, 1–8. doi: 10.1038/s41467-018-07826-2, 30602718 PMC6315057

[ref8] Conover W. J. Iman R. L. (1979). Multiple-comparisons procedures. Informal report. LA-7677-MS. Los Alamos, NM (United States): Los Alamos National Lab. (LANL).

[ref9] Cruz J. Martín D. Franch-Marro X. (2020). Egfr signaling is a Major regulator of ecdysone biosynthesis in the Drosophila prothoracic gland. Curr. Biol. 30, 1547–1554.e4. doi: 10.1016/j.cub.2020.01.092, 32220314

[ref10] Debrah A. Y. Mand S. Marfo-Debrekyei Y. Batsa L. Pfarr K. Buttner M. . (2007). Macrofilaricidal effect of 4 weeks of treatment with doxycycline on *Wuchereria* bancrofti. Trop. Med. Int. Health 12, 1433–1441. doi: 10.1111/j.1365-3156.2007.01949.x, 18076549

[ref11] Debrah A. Y. Mand S. Specht S. Marfo-Debrekyei Y. Batsa L. Pfarr K. . (2006). Doxycycline reduces plasma VEGF-C/sVEGFR-3 and improves pathology in lymphatic Filariasis. PLoS Pathog. 2:e92. doi: 10.1371/journal.ppat.0020092, 17044733 PMC1564427

[ref12] Dunn O. J. (1961). Multiple comparisons among means. J. Am. Stat. Assoc. 56, 52–64. doi: 10.1080/01621459.1961.10482090

[ref13] Esner M. Graifer D. Lleonart M. E. Lyakhovich A. (2017). Targeting Cancer cells through antibiotics-induced mitochondrial dysfunction requires autophagy inhibition. Cancer Lett. 384, 60–69. doi: 10.1016/j.canlet.2016.09.023, 27693455

[ref14] Fattouh N. Cazevieille C. Landmann F. (2019). Wolbachia endosymbionts subvert the endoplasmic reticulum to acquire host membranes without triggering ER stress. PLoS Negl. Trop. Dis. 13:e0007218. doi: 10.1371/journal.pntd.0007218, 30893296 PMC6426186

[ref15] Fenollar F. Maurin M. Raoult D. (2003). *Wolbachia* pipientis growth kinetics and susceptibilities to 13 antibiotics determined by immunofluorescence staining and real-time PCR. Antimicrob. Agents Chemother. 47, 1665–1671. doi: 10.1128/AAC.47.5.1665-1671.2003, 12709338 PMC153309

[ref16] Foster J. Ganatra M. Kamal I. Ware J. Makarova K.. (2005). The Wolbachia genome of Brugia Malayi: endosymbiont evolution within a human pathogenic nematode. PLoS Biol. 3:e121. doi: 10.1371/journal.pbio.0030121 15780005 PMC1069646

[ref17] Geoghegan V. Stainton K. Rainey S. M. Ant T. H. Dowle A. A. Larson T. . (2017). Perturbed cholesterol and vesicular trafficking associated with dengue blocking in Wolbachia-infected *Aedes Aegypti* cells. Nat. Commun. 8:1. doi: 10.1038/s41467-017-00610-8, 28904344 PMC5597582

[ref18] Griffiths K. G. Alworth L. C. Harvey S. B. Michalski M. L. (2010). Using an intravenous catheter to carry out abdominal lavage in the gerbil. Lab Anim. 39, 143–148. doi: 10.1038/laban0510-143 20410898

[ref19] Hargitai D. Kenéz L. Al-Lami M. Szenczi G. Lőrincz P. Juhász G. (2022). Autophagy controls Wolbachia infection upon bacterial damage and in aging Drosophila. Front. Cell Dev. Biol. 10:976882. doi: 10.3389/fcell.2022.976882, 36299486 PMC9589277

[ref20] Hermans P. G. Hart C. A. Trees A. J. (2001). In vitro activity of antimicrobial agents against the endosymbiont Wolbachia Pipientis. J. Antimicrob. Chemother. 47, 659–663. doi: 10.1093/jac/47.5.659, 11328780

[ref21] Hoerauf A. Specht S. Büttner M. Pfarr K. Mand S. Fimmers R. . (2008). Wolbachia Endobacteria depletion by doxycycline as Antifilarial therapy has Macrofilaricidal activity in onchocerciasis: a randomized placebo-controlled study. Med. Microbiol. Immunol. 197, 295–311. doi: 10.1007/s00430-007-0072-z, 17999080 PMC2668626

[ref22] Hoerauf A. Volkmann L. Nissen Paehle K. Schmetz C. Autenrieth I. Büttner D. W. . (2000). Targeting of Wolbachia Endobacteria in Litomosoides Sigmodontis: comparison of Tetracyclines with chloramphenicol, macrolides and ciprofloxacin. Trop. Med. Int. Health 5, 275–279. doi: 10.1046/j.1365-3156.2000.00544.x 10810023

[ref23] Hong W. D. Benayoud F. Nixon G. L. Ford L. Johnston K. L. Clare R. H. . (2019). AWZ1066S, a highly specific anti-Wolbachia drug candidate for a short-course treatment of Filariasis. Proc. Natl. Acad. Sci. USA 116, 1414–1419. doi: 10.1073/pnas.1816585116, 30617067 PMC6347715

[ref24] Johnston K. L. Cook D. A. Berry N. G. David Hong W. Clare R. H. Goddard M. . (2017). ‘Identification and prioritization of novel anti-Wolbachia Chemotypes from screening a 10,000-compound diversity library’. *Science*. Advances 3:eaao1551. doi: 10.1126/sciadv.aao1551 PMC5617373 28959730

[ref25] Johnston K. L. Ford L. Umareddy I. Townson S. Specht S. Pfarr K. . (2014). Repurposing of approved drugs from the human pharmacopoeia to target *Wolbachia* endosymbionts of onchocerciasis and lymphatic Filariasis. Int. J. Parasitol. Drugs Drug Resist., Includes articles from two meetings: ‘Anthelmintics: From Discovery to Resistance’, 218–315, and ‘Global Challenges for New Drug Discovery Against Tropical Parasitic Diseases, 316–357 4, 278–286. doi: 10.1016/j.ijpddr.2014.09.001 25516838 PMC4266796

[ref26] Kim J.-J. Lee H.-M. Shin D.-M. Kim W. Yuk J. M. Jin H. S. . (2012). Host cell autophagy activated by antibiotics is required for their effective Antimycobacterial drug action. Cell Host Microbe 11, 457–468. doi: 10.1016/j.chom.2012.03.008, 22607799

[ref27] Klionsky D. J. Abdel-Aziz A. K. Abdelfatah S. Abdellatif M. Abdoli A. Abel S. . (2021). Guidelines for the use and interpretation of assays for monitoring autophagy. Autophagy 17, 1–382. doi: 10.1080/15548627.2020.1797280, 33634751 PMC7996087

[ref28] Kruskal W. H. Wallis W. A. (1952). Use of ranks in one-criterion variance analysis. J. Am. Stat. Assoc. 47, 583–621. doi: 10.1080/01621459.1952.10483441

[ref29] Levine B. Kroemer G. (2019). Biological functions of autophagy genes: a disease perspective. Cell 176, 11–42. doi: 10.1016/j.cell.2018.09.048, 30633901 PMC6347410

[ref30] McGarry H. F. Egerton G. L. Taylor M. J. (2004). Population dynamics of Wolbachia bacterial endosymbionts in Brugia Malayi. Mol. Biochem. Parasitol. 135, 57–67. doi: 10.1016/j.molbiopara.2004.01.006, 15287587

[ref31] Mizushima N. (2007). Autophagy: process and function. Genes Dev. 21, 2861–2873. doi: 10.1101/gad.1599207, 18006683

[ref32] Nagy T. A. Quintana J. L. J. Reens A. L. Crooks A. L. Detweiler C. S. (2019). Autophagy induction by a small molecule inhibits *Salmonella* survival in macrophages and mice. Antimicrob. Agents Chemother. 63:10.1128/aac.01536-19. doi: 10.1128/aac.01536-19, 31591121 PMC6879225

[ref33] O’Neill S. L. Pettigrew M. M. Sinkins S. P. Braig H. R. Andreadis T. G. Tesh R. B. (1997). In vitro cultivation of Wolbachia Pipientis in an *Aedes Albopictus* cell line. Insect Mol. Biol. 6, 33–39. doi: 10.1046/j.1365-2583.1997.00157.x, 9013253

[ref34] Parzych K. R. Klionsky D. J. (2014). An overview of autophagy: morphology, mechanism, and regulation. Antioxid. Redox Signal. 20, 460–473. doi: 10.1089/ars.2013.5371, 23725295 PMC3894687

[ref35] Peyrusson F. Varet H. Nguyen T. K. Legendre R. Sismeiro O. Coppée J. Y. . (2020). Intracellular *Staphylococcus Aureus* Persisters upon antibiotic exposure. Nat. Commun. 11:1. doi: 10.1038/s41467-020-15966-7, 32366839 PMC7198484

[ref36] Powis G. Bonjouklian R. Berggren M. M. Gallegos A. Abraham R. Ashendel C. . (1994). Wortmannin, a potent and selective inhibitor of phosphatidylinositol-3-kinase. Cancer Res. 54, 2419–2423. 8162590

[ref37] Quek S. Cook D. A. N. Wu Y. Marriott A. E. Steven A. Johnston K. L. . (2022). *Wolbachia* depletion blocks transmission of lymphatic filariasis by preventing chitinase-dependent parasite exsheathment. Proc. Natl. Acad. Sci. USA 119:e2120003119. doi: 10.1073/pnas.2120003119, 35377795 PMC9169722

[ref38] Rasgon J. L. Gamston C. E. Ren X. (2006). Survival of Wolbachia Pipientis in cell-free medium. Appl. Environ. Microbiol. 72, 6934–6937. doi: 10.1128/AEM.01673-06, 16950898 PMC1636208

[ref39] Royston J. P. (1982). Algorithm AS 181: the W test for normality. Appl. Stat. 31, 176–180. doi: 10.2307/2347986

[ref40] Rusten T. E. Lindmo K. Juhász G. Sass M. Seglen P. O. Brech A. . (2004). Programmed autophagy in the Drosophila fat body is induced by ecdysone through regulation of the PI3K pathway. Dev. Cell 7, 179–192. doi: 10.1016/j.devcel.2004.07.005, 15296715

[ref41] Sharma R. Al Jayoussi G. Tyrer H. E. Al Jayoussi G. Gamble J. Hayward L. . (2016). Minocycline as a re-purposed anti-*Wolbachia* macrofilaricide: superiority compared with doxycycline regimens in a murine infection model of human lymphatic filariasis. Sci. Rep. 6:23458. doi: 10.1038/srep23458, 26996237 PMC4800446

[ref42] Slatko B. E. Taylor M. J. Foster J. M. (2010). The *Wolbachia* endosymbiont as an anti-filarial nematode target. Symbiosis 51, 55–65. doi: 10.1007/s13199-010-0067-1, 20730111 PMC2918796

[ref43] Specht S. Mand S. Marfo-Debrekyei Y. Debrah A. Y. Konadu P. Adjei O. . (2008). Efficacy of 2- and 4-week rifampicin treatment on the Wolbachia of Onchocerca volvulus. Parasitol. Res. 103, 1303–1309. doi: 10.1007/s00436-008-1133-y, 18679718

[ref44] Stanley S. A. Barczak A. K. Silvis M. R. Luo S. S. Sogi K. Vokes M. . (2014). Identification of host-targeted small molecules that restrict intracellular *Mycobacterium Tuberculosis* growth. PLoS Pathog. 10:e1003946. doi: 10.1371/journal.ppat.1003946, 24586159 PMC3930586

[ref45] Taylor M. J. Bandi C. Hoerauf A. (2005a). *Wolbachia*. Bacterial endosymbionts of filarial nematodes. Adv. Parasitol. 60, 245–284. doi: 10.1016/S0065-308X(05)60004-8 16230105

[ref46] Taylor M. J. Hoerauf A. Bockarie M. (2010). Lymphatic filariasis and onchocerciasis. Lancet 376, 1175–1185. doi: 10.1016/s0140-6736(10)60586-7, 20739055

[ref47] Taylor M. J. Hoerauf A. Townson S. Slatko B. E. Ward S. A. (2013a). Anti-Wolbachia drug discovery and development: safe Macrofilaricides for onchocerciasis and lymphatic Filariasis. Parasitology 141, 119–127. doi: 10.1017/S0031182013001108, 23866958 PMC3884836

[ref48] Taylor M. J. Makunde W. H. McGarry H. F. Turner J. D. Mand S. Hoerauf A. (2005b). Macrofilaricidal activity after doxycycline treatment of Wuchereria Bancrofti: a double-blind, randomised placebo-controlled trial. Lancet 365, 2116–2121. doi: 10.1016/s0140-6736(05)66591-9, 15964448

[ref49] Taylor M. J. Voronin D. Johnston K. L. Ford L. (2013b). Wolbachia filarial interactions. Cell. Microbiol. 15, 520–526. doi: 10.1111/cmi.12084, 23210448

[ref50] Townson S. Tagboto S. McGarry H. F. Egerton G. L. Taylor M. J. (2006). Onchocerca parasites and Wolbachia endosymbionts: evaluation of a Spectrum of antibiotic types for activity against Onchocerca Gutturosa in vitro. Filaria J. 5, 1–9. doi: 10.1186/1475-2883-5-4 16563157 PMC1471782

[ref51] Turner J. D. Stuart Langley R. Johnston K. L. Egerton G. Wanji S. Taylor M. J. (2006). Wolbachia endosymbiotic Bacteria of Brugia Malayi mediate macrophage tolerance to TLR- and CD40-specific stimuli in a MyD88/TLR2-dependent Manner1. J. Immunol. 177, 1240–1249. doi: 10.4049/jimmunol.177.2.1240, 16818783

[ref52] Voronin D. Cook D. A. N. Steven A. Taylor M. J. (2012). Autophagy regulates Wolbachia populations across diverse symbiotic associations. Proc. Natl. Acad. Sci. USA 109, E1638–E1646. doi: 10.1073/pnas.1203519109, 22645363 PMC3382551

[ref53] Voronin D. Tricoche N. Peguero R. Kaminska A. M. Ghedin E. Sakanari J. A. . (2024). Repurposed drugs that activate autophagy in filarial Worms act as effective Macrofilaricides. Pharmaceutics 16:2. doi: 10.3390/pharmaceutics16020256, 38399310 PMC10891619

[ref54] Wickham Hadley Sievert Carson (2009) Ggplot2: Elegant graphics for data analysis 10 Springer New York. Available online at: https://academic.oup.com/biometrics/article-abstract/67/2/678/7381027?login=false (Accessed December 10, 2025).

[ref55] World Health Organization (2017) Guideline: Alternative mass drug administration regimens to eliminate lymphatic Filariasis World Health Organization. Available online at: https://apps.who.int/iris/handle/10665/259381 (Accessed January 12, 2025). 29565523

[ref56] World Health Organization (2025) Global Programme to eliminate lymphatic Filariasis: Progress report, 2024. Available online at: https://www.who.int/publications/i/item/who-wer10040-439-449 (Accessed January 12, 2025).

[ref57] Xiao Y. Xiong T. Meng X. Yu D. Xiao Z. Song L. (2019). Different influences on mitochondrial function, oxidative stress and cytotoxicity of antibiotics on primary human neuron and cell lines. J. Biochem. Mol. Toxicol. 33:e22277. doi: 10.1002/jbt.22277, 30597674

[ref58] Yandell B. S. (2017). Practical data analysis for designed experiments. Boca Raton, FL: Chapman & Hall/CRC Taylor & Francis Group.

[ref59] Yang Y.-p. Hu L.-f. Zheng H.-f. Mao C.-j. Hu W.-d. Xiong K.-p. . (2013). Application and interpretation of current autophagy inhibitors and activators. Acta Pharmacol. Sin. 34, 625–635. doi: 10.1038/aps.2013.5, 23524572 PMC4002883

