# Supplementary material for: Autophagy is essential for anti-Wolbachia drug efficacy in Brugia malayi and insect cells
Source: Front Microbiol. 2026 Mar 16;17:1771755. doi: 10.3389/fmicb.2026.1771755 (PMC13033743; doi:10.3389/fmicb.2026.1771755)
Supplement: Supplementary file 1 [file Table_1.DOCX]

**
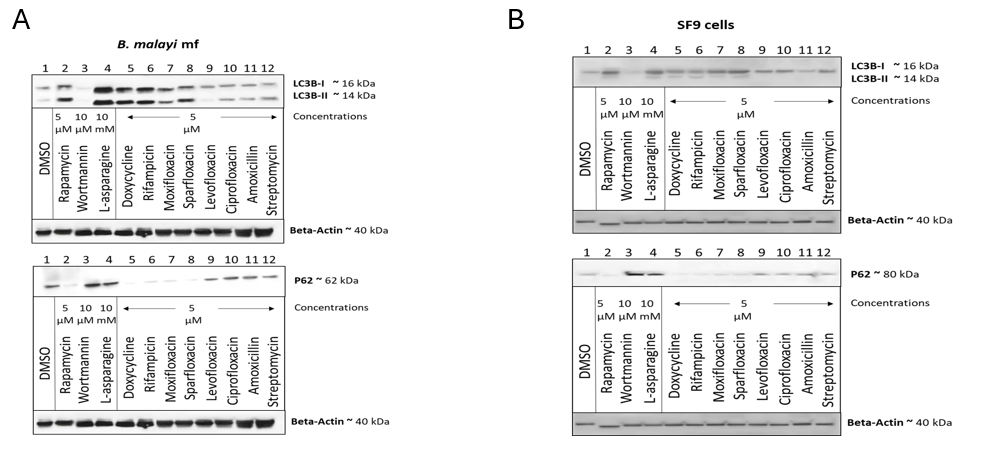
**

**
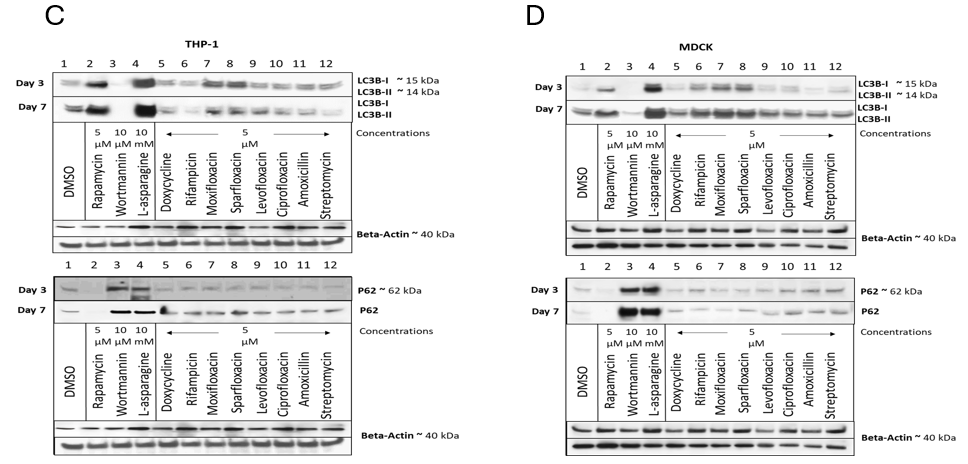
**

**Figure S1. Detection of LC3B and p62 expression for antibiotics from diverse classes in SF9 cells, *Brugia malayi* mf, THP-1 and MDCK cells using western blot.**

A) *B. malayi* mf worms, B) SF9 insect cells, C) THP-1 cells and D) MDCK cells were treated with: DMSO – vehicle control (lane 1), rapamycin – positive control (lane 2), wortmannin – early autophagy inhibitor (lane 3), l-asparagine – late autophagy inhibitor (lane 4), four anti-*Wolbachia* agents: doxycycline (lane 5), rifampicin (lane 6), moxifloxacin (lane 7), sparfloxacin (lane 8), and four different antibiotics: levofloxacin (lane 9), ciprofloxacin (lane 10), amoxicillin (lane 11), and streptomycin (lane 12) for 3 days. Reduced protein extracts of cells were loaded at 50 μg/40 µl per lane into 4-12% bis tris SDS-PAGE gels. Separated proteins were transferred into nitrocellulose membrane, blocked and incubated with primary and secondary antibodies. Western blot protein expression is presented in for rabbit anti-LC3B (LC3B-I at 16 kDa and LC3B-II at 14 kDa) and rabbit anti-p62 (at 80 kDa for SF9 and 62 kDa for mf). A reference protein; mouse anti-beta actin was used as a control with a size of approximately 40 kDa**.**

**
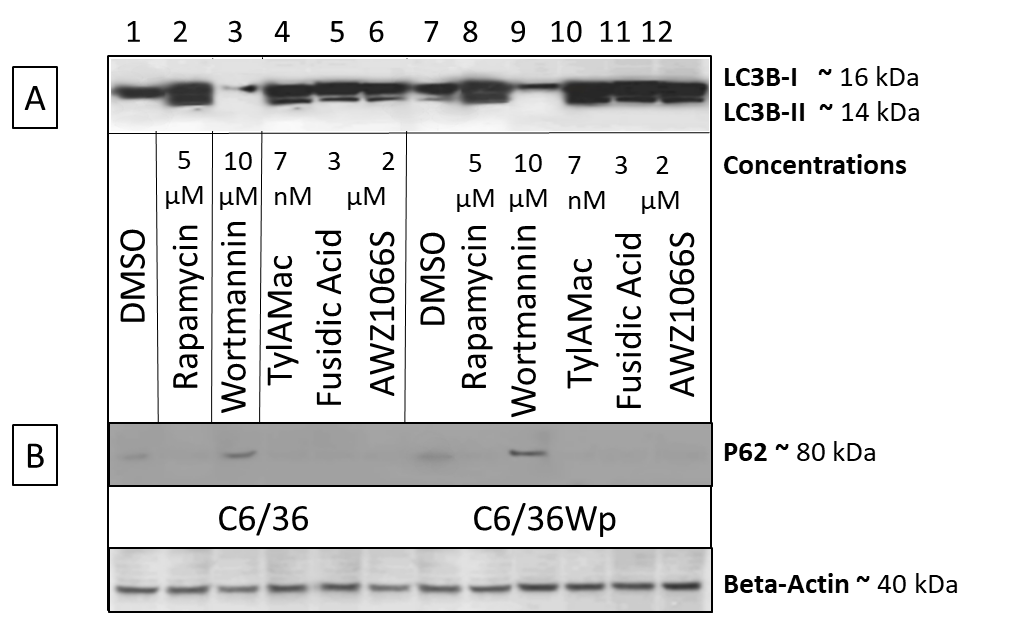
**

**Figure S2. Detection of LC3B and p62 expression for A-WOL selected candidates and repurposed anti-*Wolbachia* agents in C6/36 cells using western blot.**

*Wolbachia*-free C6/36 (lane 1-6) and infected C6/36*Wp* cells (lane 7-12) were treated with: DMSO – vehicle control (lane 1 and 7), rapamycin – positive control (lane 2 and 8), wortmannin – autophagy inhibitor (lane 3 and 9), TylAMacTM (lane 4 and 10), fusidic acid (lane 5 and 11), and AWZ1066S (lane 6 and 12) for 3 days. Reduced protein extracts of cells were loaded at 50 μg/40 ul per lane into 4-12% bis tris SDS-PAGE gels. Separated proteins were transferred into nitrocellulose membrane, blocked and incubated with primary and secondary antibodies. Western blot protein expression is presented in A) for rabbit anti-LC3B (LC3B-I at 16 kDa and LC3B-II at 14 kDa) and B) rabbit anti-p62 (at 80 kDa). A reference protein; mouse anti-beta actin was used as a control with a size of approximately 40 kDa.


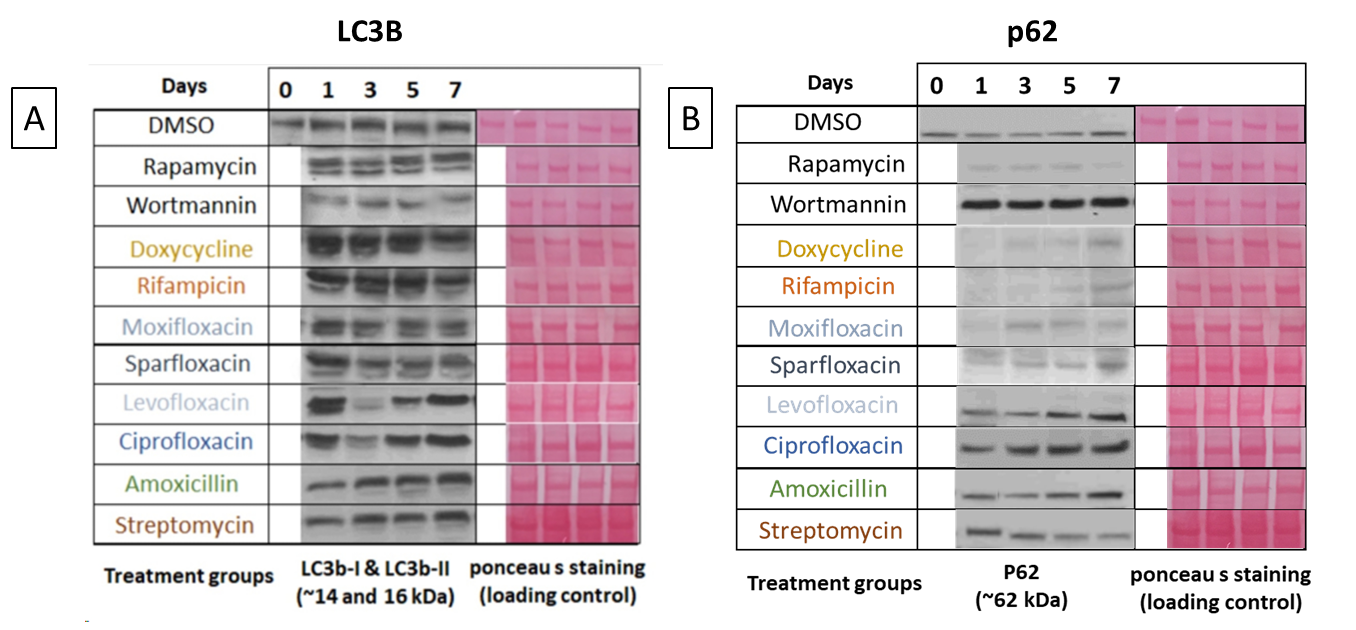


**Figure S3. Immunoblotting analysis for autophagy induction at different time-points using diverse antibiotics in C6/36*Wp* cells.**

C6/36 mosquito cells infected with *Wolbachia* (*w*AlbB) treated with: DMSO – vehicle control, rapamycin – positive control, wortmannin -autophagy inhibitor, four anti-*Wolbachia* agents: doxycycline, rifampicin, moxifloxacin, sparfloxacin, and four different antibiotics: levofloxacin, ciprofloxacin, amoxicillin, and streptomycin at different time-points: day 0, 1, 3, 5 and 7. Reduced protein extracts of cells were loaded at 50μg/40ul per lane into 4-12% bis tris SDS- PAGE gels. Separated proteins were transferred into nitrocellulose membrane, blocked and incubated with primary and secondary antibodies. Western blot protein expression is presented in A) for rabbit anti-LC3B (LC3B-I at 16 kDa and LC3B-II at 14 kDa) and B) rabbit anti-p62 (at 80 kDa). Ponceau s staining was used as a loading control.


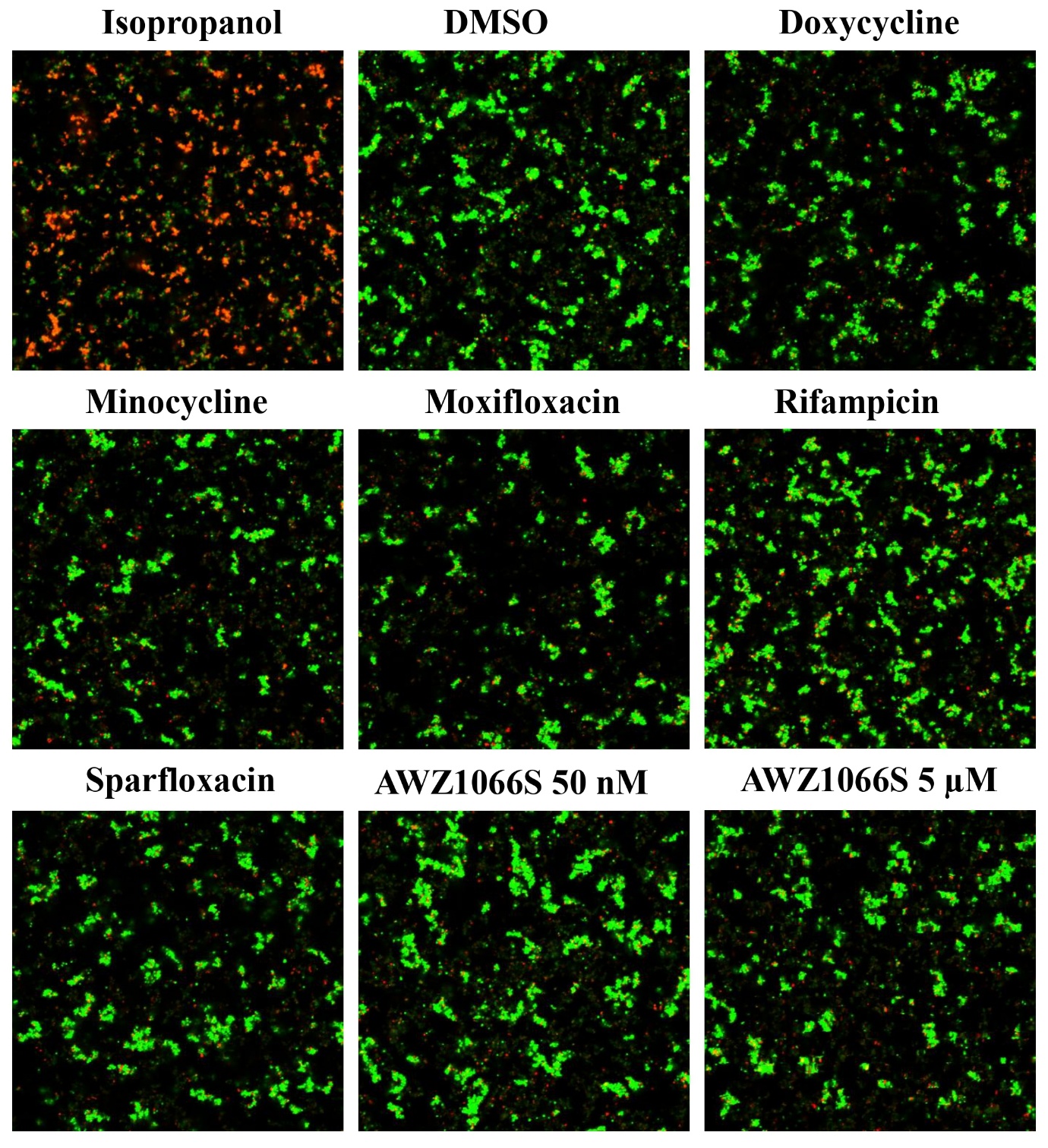


**Figure S4. Representative images of BacLight staining after drug treatment of extracellular *Wolbachia*.** *Wolbachia* were purified from C6/36 cells and incubated with 5 μM of each drug for 7-days in a black-sided 96-well plate. Bacteria were stained with BacLight staining and imaged on a confocal microscope at 63x magnification. Killed control *Wolbachia* were incubated in 70% isopropanol for 1 hour. Red indicates dead bacteria, green indicates live bacteria.
